# The Secret Life of a Lost Guidewire

**DOI:** 10.1016/j.jaccas.2025.104181

**Published:** 2025-07-23

**Authors:** Samuel McGrath, Timothy Bagnall, Nicholas Pegge, David Hildick-Smith

**Affiliations:** a King's BHF Centre of Research Excellence, King's College London, London, United Kingdom; b Sussex Cardiac Centre, Brighton and Sussex University Hospitals, Brighton, United Kingdom; c Worthing Hospital, University Sussex Hospitals, Worthing, United Kingdom

**Keywords:** complication, CT, guidewire, patent foramen ovale

## Abstract

**Background:**

Lost guidewires during central venous catheterization are rare but serious, requiring prompt removal to reduce morbidity and mortality.

**Case Summary:**

A 58-year-old woman presented with a pulsating back mass and a spontaneous bruise under her left clavicle. More than 10 years ago, a 0.035-inch guidewire had been lost in her circulation, and retrieval attempts had failed. Computed tomography scan revealed a fragmented guidewire that had crossed into the arterial circulation through a patent foramen ovale and extended through the anterior mediastinum. After a discussion among the heart team, the decision was made to remove the wire percutaneously.

**Discussion:**

This case highlights the risks of guidewire loss and the successful use of advanced catheter techniques for complex wire retrieval.

**Take-Home Messages:**

Iatrogenic guidewire loss is associated with significant risk of morbidity and mortality, and the guidewire must be removed. A fragmented wire can be removed percutaneously by forming a free loop with an additional wire. If wire embolization is recognized during central venous catheter insertion, several bedside techniques can be attempted to rapidly correct the complication.

## Visual Summary

**Figure:**
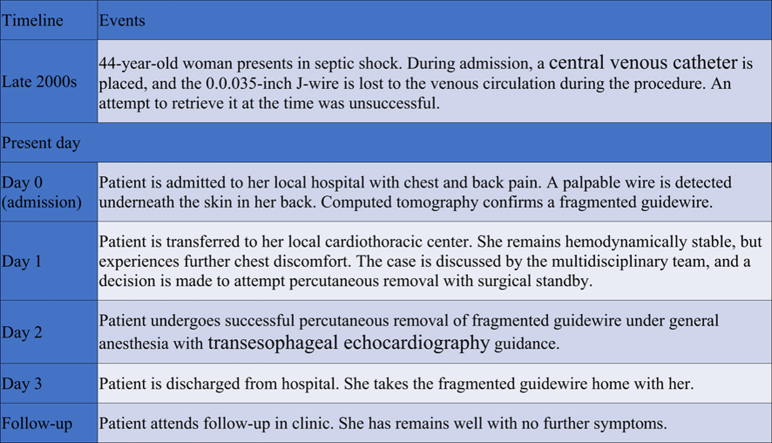

## History of Presentation

A 58-year-old woman presented to her local hospital with sharp chest pain, back pain, and bruising. Findings on examination were bruising under the left clavicle and a swelling in the right lower back where there was visible movement with each heartbeat. A palpable wire was detected underneath her skin in her right lower back. She was transferred to the cardiothoracic center for review.

**Take-Home Messages**

• Iatrogenic guidewire loss is associated with significant risk of morbidity and mortality and must be removed.
• A fragmented wire can be removed percutaneously by forming a free loop with an additional wire.
• If wire embolization is recognized during central venous catheter insertion, several bedside techniques can be attempted to rapidly correct the complication.

## Past Medical History

The patient had a history of ulcerative colitis. In 2008, she was admitted to the hospital in septic shock, and this required a short admission to the intensive care unit. During this admission, a central venous catheter was placed via a femoral vein. A 0.035-inch J-wire was lost to the venous circulation. The patient reported that an attempt to retrieve it at the time was unsuccessful. The written notes from the central venous catheter insertion and attempted removal were not available for review.

## Differential Diagnosis

Based on the events in her history and her clinical examination, the suspected diagnosis was migration and possible fragmentation of the 0.035-inch guidewire.

## Investigations

**Figure 1:**
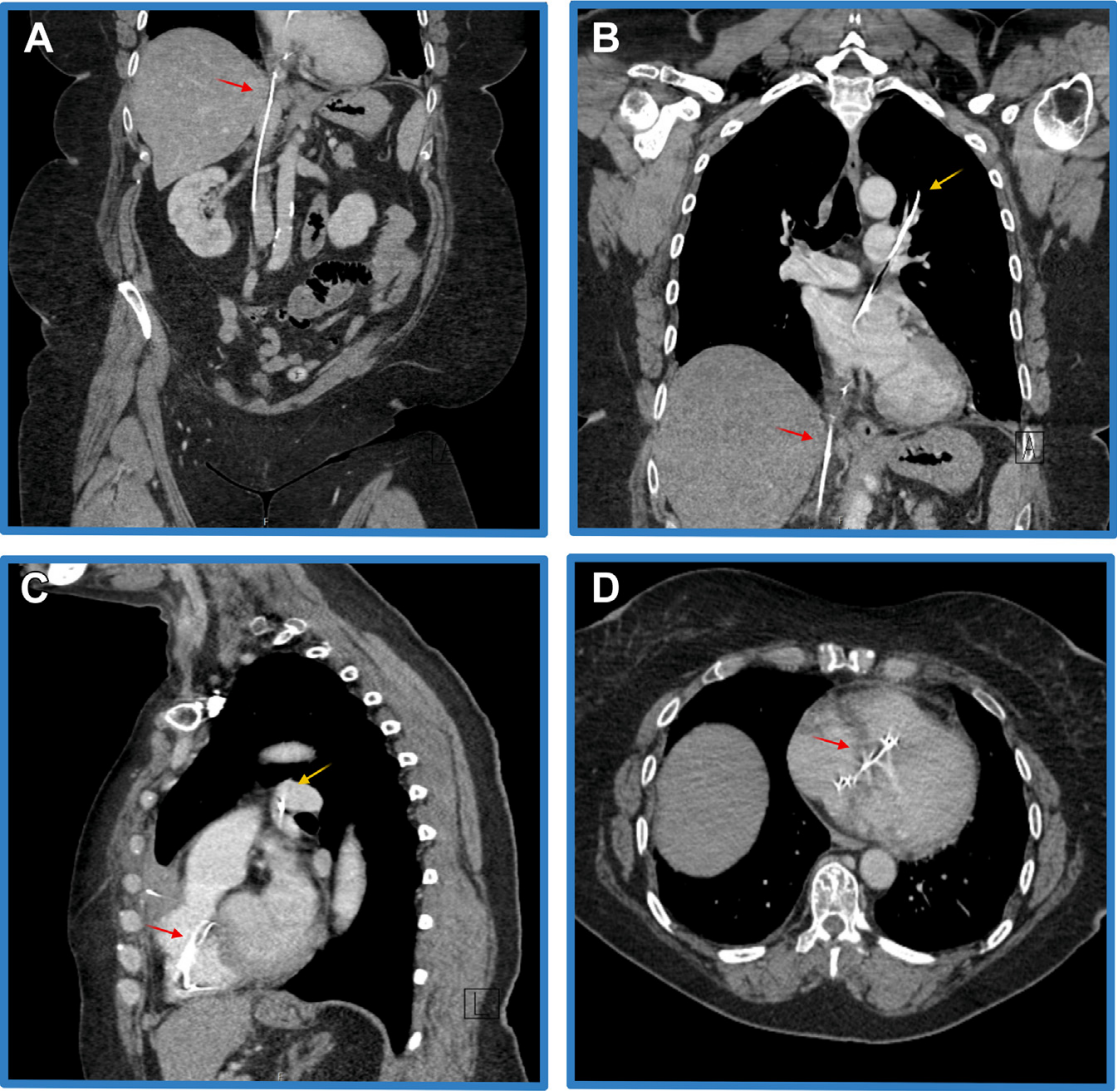
Intravascular Course of the Wire, With 4 Separate Still Images Taken From Computed Tomography of Different Slices Transverse thorax slice showing the wire crossing the atrial septum (red arrow). (A) Coronal abdominal slice. Red arrow indicates a wire within the inferior vena cava extending up into the right atrium. (B) Coronal thorax slice. Red arrow shows the wire entering the heart via the inferior vena cava, traversing the septum, and reaching the upper left pulmonary vein (yellow arrow). (C) Sagittal thorax slice. Red arrow highlights filaments of the unraveled wire within the right ventricle. Yellow arrow indicates the wire within the pulmonary venous vasculature, again confirming it has crossed the septum. (D) Transverse thorax slice. This view clearly shows the wire crossing the atrial septum.

**Figure 2:**
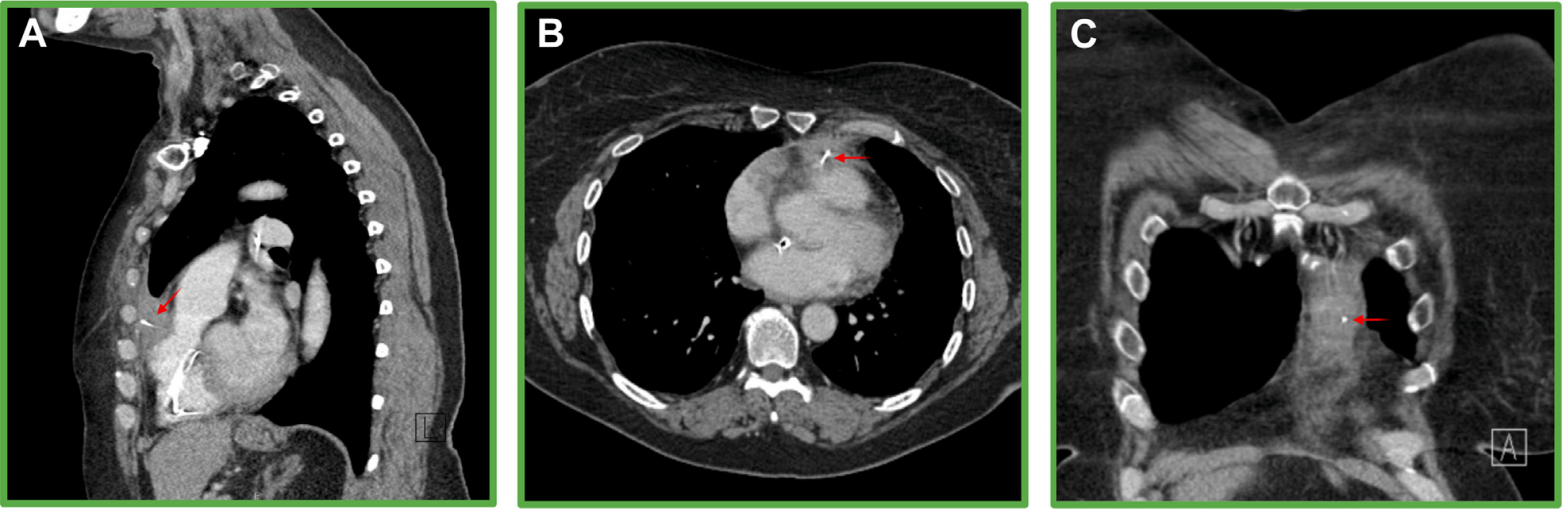
Anterior Course of the Wire, With 3 Separate Still Images Taken From Computed Tomography of Different Slices In each respective view, the red arrow details the course of the wire within the thorax. (A) Sagittal thorax slice. The wire can be seen coursing through the right ventricular outflow tract through the pericardium, through the subcutaneous layer of chest wall coursing superiorly to lie in the left anterior superior chest wall. (B) Transverse thorax slice. The exit point of the wire from the right ventricular outflow tract into the pericardium. (C) Coronal thorax slice. This view helps visualize how high the final position of the wire is in the left anterior chest wall.

**Figure 3:**
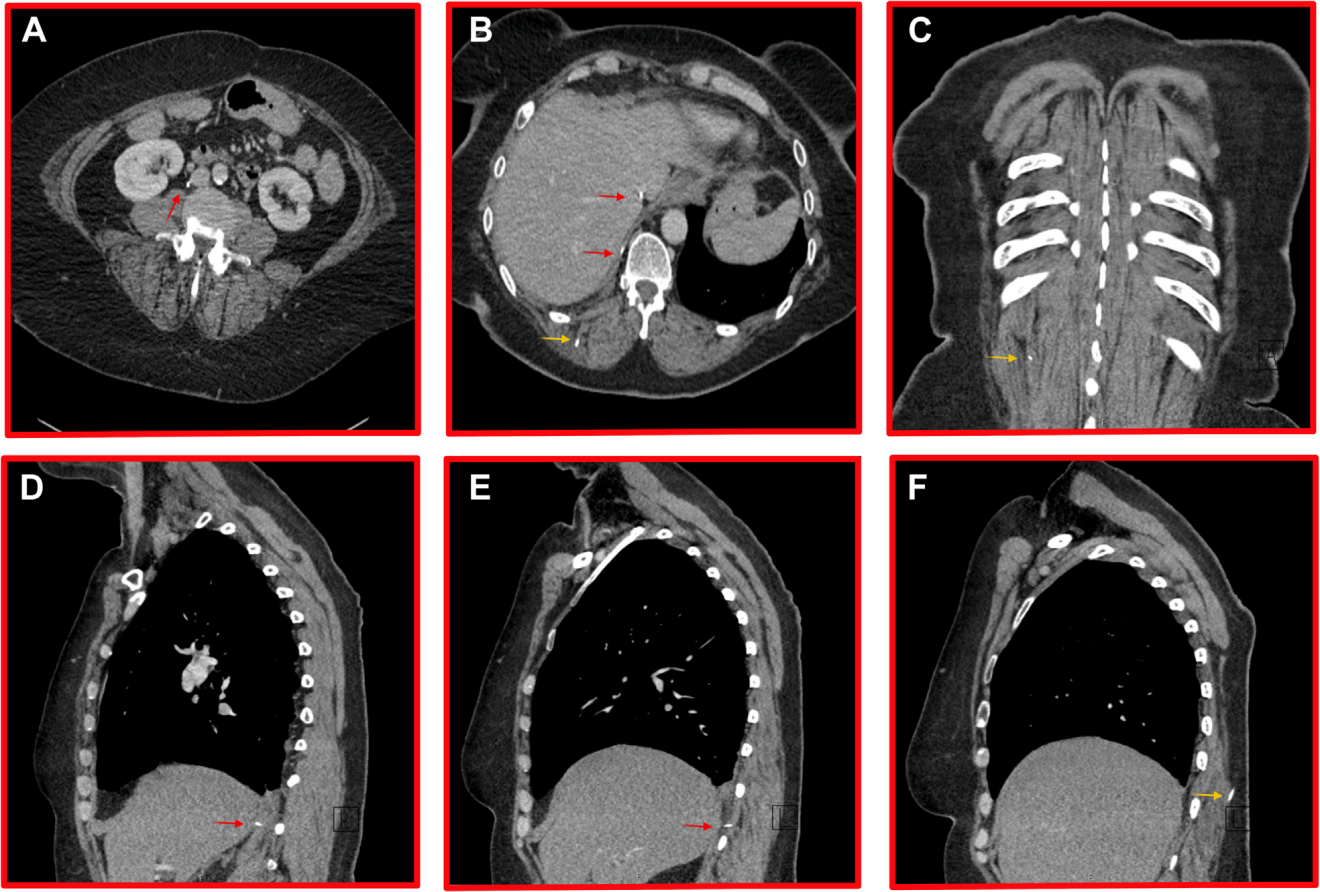
Posterior Course of the Wire, With 6 Separate Still Images Taken From Computed Tomography of Different Slices In each respective view, the red arrow details the cause of the wire within the abdomen, and the yellow arrow details the extravascular course within muscle or subcutaneous tissue. (A) Transverse thorax slice. The wire is seen exiting the heart posteriorly into the posterior basal segment of the right lower lobe. The exit point is thought to be the junction of the inferior vena cava and the right atrium. (B) Transverse thorax slice. The wire has gone beneath the diaphragm. (C) Coronal thorax slice. The exit point is via the right paravertebral soft tissues adjacent to T12. (D) Sagittal thorax slice. This view shows the wire traveling posteriorly. (E) Sagittal thorax slice. The wire is seen exiting adjacent to T12. (F) Sagittal thorax slice. The wire is seen traveling through the soft tissues to just beneath the skin.

A computed tomography scan showed the main body of the wire within the right atrium extending across a presumed patent foramen ovale into the left atrium and up into the upper left pulmonary vein. There was also an unraveled filament traveling into the right ventricle and further wire traveling down the inferior vena cava. There appeared to be a separate fragment of wire, which traversed the anterior mediastinum, was partially intravascular, and then ran extravascularly under the diaphragm and to the skin in the patient's back. The intravascular course of the wire can be seen in Figures 1A to 1D. The anterior and posterior course of the wire are demonstrated in Figures 2A to 2C and Figures 3A to 3F respectively. Video 1 demonstrates a three-dimensional reconstruction of the course of the wire.

## Management

Given the presentation and risk of wire erosion, infection, or stroke, members of the multidisciplinary heart team agreed that the wires should be removed. The consensus was that this should be attempted percutaneously with surgical standby in case of irretrievability or bleeding complications.

### Intervention

The procedure was performed under general anesthesia with fluoroscopy and transesophageal echocardiography guidance. Cardiopulmonary support was available, and cardiac surgeons were present. An equipment list for the procedure is shown in Table 1. Right femoral vein access was obtained. The vein was occluded in its iliac course, presumably in relation to the presence of the wire, but this was recanalized with a Terumo wire into the inferior vena cava, allowing access to the heart. Access was switched to the left femoral vein with a 16-F sheath, as the initial access site was heavily restrictive and fibrosed. A cerebral embolic protection filter was placed via the right radial artery. The case was predominantly guided by fluoroscopy, and transesophageal echocardiography was used to confirm the course of the wire within the heart (Video 2).

**Table 1:** Equipment List for Procedure

Imaging • Tranesophageal echocardiography (TEE) (Philips Healthcare, USA) ○ X7-2t TEE probe
Access • Ultrasound machine (Philips Healthcare, USA) • 0.035 J wire and 16-F sheath • Proglide (Abbott Vascular, USA)
Procedure • Short curved (16.8 mm) Agilis 8.5F steerable introducer (Abbott Vascular, USA) • Long 0.035 J wire • Long 0.035 Terumo Wire (Terumo, USA) • 6-F pigtail catheter • Goose neck snare (Medtronic, USA)

**Figure 4:**
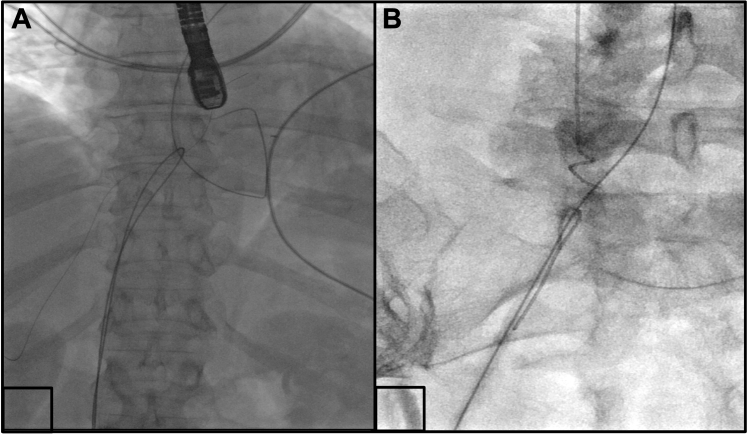
Exteriorized Wire Looping Around the Fragmented Wire (A) An exteriorized wire being looped around the fragment within the right atrium. (B) The gooseneck snare coming out of the Agilis catheter to snare the free end of the wire.

There was no free end of wire available for snaring; therefore a short-curved Agilis steerable sheath was used to hook over the right atrial segment of 0.035-inch wire. An exchange length 0.035-inch wire was passed around the lost wire through the Agilis and snared in the lower inferior vena cava. This was exteriorized, creating a wire loop (Video 3, Figure 4). This wire loop was used to invaginate the lost wire into the Agilis sheath and remove it (Video 4). The wire could be seen passing down from the left upper pulmonary vein, to the left atrium, through the patent foramen ovale, and then into the right atrium. The wire fragmented on removal and a 4-cm intact segment remained adherent to the right ventricle where it was not safely accessible and was left in situ. Echocardiographic images suggested that it was strongly adherent to the right ventricular wall.

The second lost wire fragment that was pulsating in her back was then targeted. This was approached using a short-curved Agilis catheter and an exchange length 0.035-inch wire in the right atrium. The wire was successfully hooked by the Agilis, and the long wire was snared and exteriorized. The wire fragment was again invaginated into the Agilis. Both the fragment that was in the mediastinum and the fragment that was under the diaphragm running to the lumbar region were removed as a single entity. Examination suggested that this part of the wire was the inner core (30-cm filament), which is inherent to the structure of the standard 0.035-inch J-wire.

## Outcome and Follow-Up

**Figure 5:**
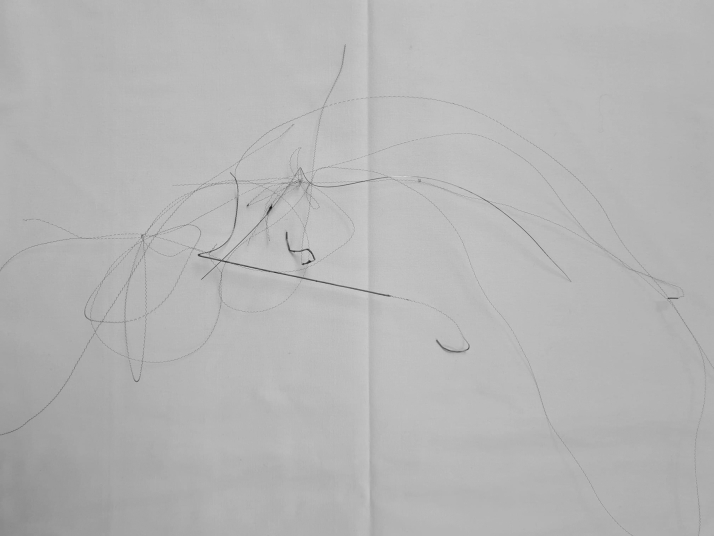
Fragmented Guidewire After Removal The Patient asked to keep the fragment after the procedure

There were no procedural complications. The patient's symptoms resolved, and she went home the next day with the wire in a bag as a memento. At a follow-up examination 6 months later, she brought the wire with her (Figure 5), and she remains well.

## Discussion

Percutaneous central venous cannulation is a common procedure. Complications include accidental arterial puncture, infection, bleeding, or bruising.^1^ The complication rate is low, especially when ultrasound guidance is used. In very rare circumstances, a guidewire can be lost in the venous circulation during the procedure, though this is a “never” event.^2^

The guidewire is composed of 2 main components made of stainless steel: an inner single filament wire core and an outer coiled wire cover. The outer cover is a helical structure, which provides elasticity and forms a central tunnel. The core wire is positioned within this tunnel, allowing for stability during medical procedures.^3^ There have been a few reports of unraveled guidewires in the literature, typically owing to damage to the wire at the time of insertion.^3^^,^^4^ The mechanism by which the guidewire unraveled in the present case is unclear; it is possible that the course the wire had taken had caused a kink, which resulted in a break in the outer cover over time.

If a guidewire is lost, this necessitates removal, as foreign body embolism carries a significant risk of morbidity or even mortality.^5^ Guidewire risks include vessel perforation, thrombosis, and infection.^6^ The longer a lost wire is retained, the higher the risk of adhesions forming with the surrounding vasculature, thereby increasing the risk of the procedure.

In our case, many of the admission notes are unavailable, leaving several unanswered questions about the clinical decisions made, the equipment used, and the sequence of events. The timing of when the lost guidewire was recognized is unclear, as are the techniques attempted for removal and the reasoning behind discontinuing further attempts. The evidence clearly supports the removal of embolized guidewires, but the absence of detailed documentation prevents a comprehensive understanding of the approach taken at the time of guidewire loss.

Several studies suggest that most cases of guidewire embolization go unnoticed until after the procedure.^2^^,^^6^ However, if recognized during insertion, several bedside techniques can be used for rapid correction. These include clamping and removing the catheter-wire unit as one or using the “suck-out” technique with a large syringe.^2^^,^^6^^,^^7^ The success of these bedside methods depends on the position of the wire relative to the central venous catheter.

If attempted at on a separate occasion, the removal of an embolized wire is usually straightforward using a loop snare such as a gooseneck snare.^8^ Before the introduction of the gooseneck snare, operators would rely on a variety of methods including dormie baskets and grasping forceps.^9^ Snares, however, require a free end of wire for grasping. If there is no free end of wire, a loop around the wire has to be created. This can be done using a needle-eye snare, or a pigtail catheter rotated around the object repeatedly to hold it firmly.^8^ This, however, creates a risk of further impaction.

**Figure 6:**
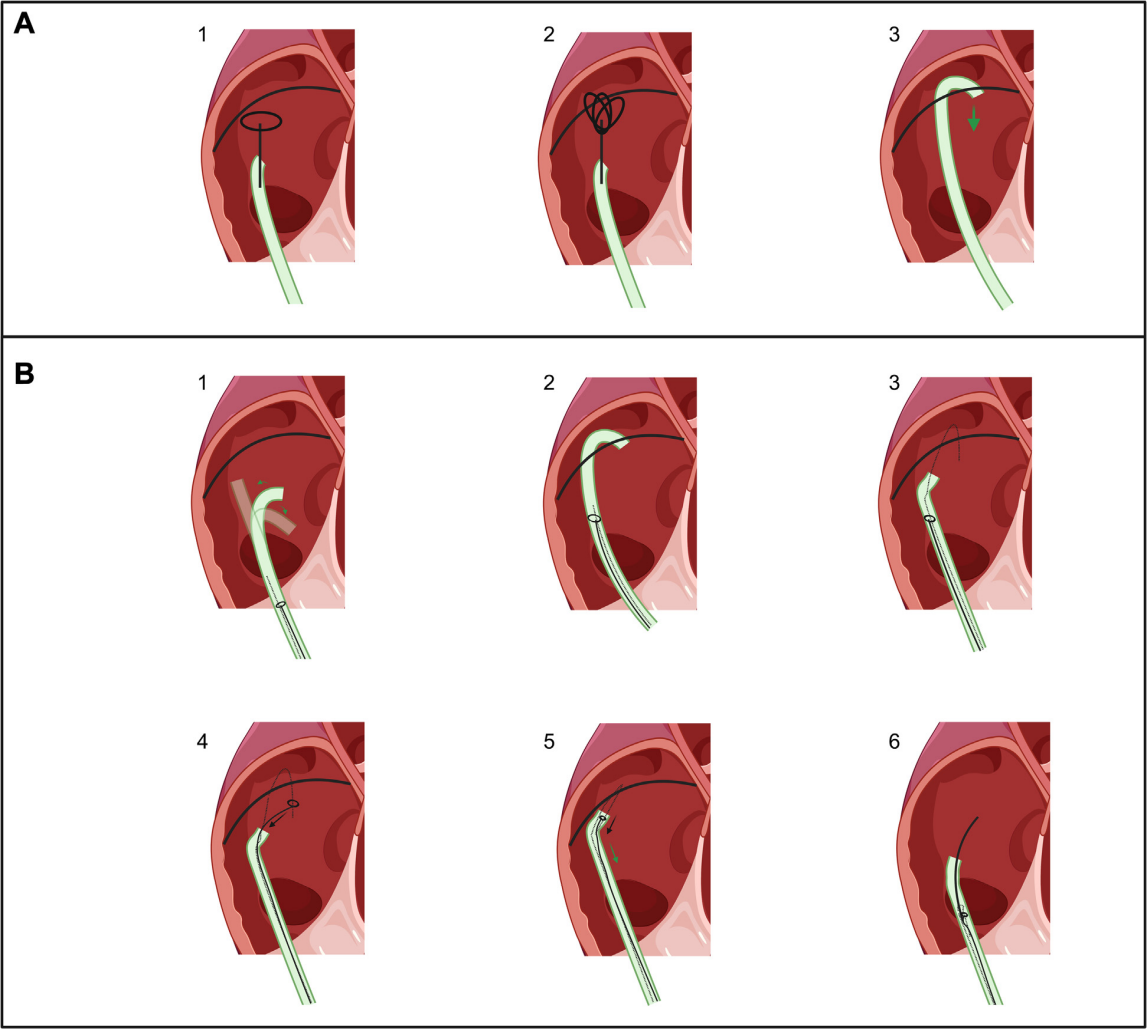
Conventional Snaring Methods and Hangman Technique (A) Failure of conventional snaring techniques. (B) Hangman snaring technique used in our case. (A) Step 1: A gooseneck snare fails to grip the wire fragment owing to the absence of a loose end. Step 2: A tulip (EN Snare) snare can grasp the wire, but lacks sufficient purchase on the fragments. Step 3: A catheter can hook over the wire, but the operator loses significant tactile feedback. This is difficult when applying force and increases the risk of injuring the surrounding structures. (B) Step 1: A steerable catheter of adequate French size is required to accommodate multiple instruments. The dotted line represents the free J-wire within the catheter, which is used to form the loop, and the gooseneck snare is also within the catheter. Step 2: The catheter is manipulated to hook around the wire. Step 3: The free J wire loops around the free fragment, and the catheter is retracted and straightened. Step 4: A gooseneck snare captures the loose end of the J wire, securing it around the fragment. Step 5: The snare is withdrawn into the catheter, creating a tight loop around the wire fragment. The operator applies traction by pulling the snare further into the catheter while maneuvering the catheter itself. Step 6: The wire fragment is successfully withdrawn into the catheter.

In the present case, the fragments of wire were firmly adherent to the right atrium and were looped to the left atrium and left upper pulmonary vein via a patent foramen ovale. We thought that the risk of vascular injury or further wire fragmentation was high. Several fragments had no loose end for a gooseneck snare to grip. We opted to use an Agilis catheter with a short curve, as this steerable catheter gives good steerable control. Exteriorizing a J-wire around the embedded fragments and then snaring the loose end of the exteriorized wire with a gooseneck snare provided us with a greater degree of control and greater understanding of the degree of traction required to remove the embedded fragments (Video 5). This technique has been referred to as the hangman technique;^10^ Figure 6 provides a summary of this technique.

The degree of traction required was judged predominantly on tactile feedback by the operator as well as looking for minute changes in the position of the fragments on fluoroscopy. Generally, minimal force was applied, favoring steady retraction using the hangman snare. Although this still was associated with some risk of injury to the wall of the atrium, the traction and countertraction afforded by the Agilis sheath helped mitigate that risk. During initial traction of the hangman snare, resistance from the heart was palpable. As a result, the wire segment adherent to the right ventricle was left in place, as the level of force required for its removal was deemed unsafe, with a high risk of tearing the myocardium.

Another important risk consideration was the potential need to withdraw the wire through multiple extravascular structures. This was a key discussion point for the members of the heart team, and the computed tomography imaging was carefully reviewed to map the course of the wire. In this case, we concluded that withdrawing the wire into vessels via established migration channels posed a reasonably low risk of major bleeding. However, given the potential for unexpected complications, surgical backup was present, and the case was performed in a hybrid operating room.

## Conclusions

This case showcases a very unusual complication of a lost guidewire, which caused complications more than a decade later and was successfully removed percutaneously.

**Visual Summary:** Timeline of the Case

Timeline	Events
Late 2000’s	A 44 year old female presents in septic shock. During the admission she has a CVC placed, and during this procedure the 0.035” J-wire is lost to the venous circulation. An attempt to retrieve it at the time was unsuccessful
Present day	
Day 0 (admission)	Admitted to her local hospital with chest and back pain. There was palpable wire underneath the skin in her back, and a CT confirms a fragmented guidewire.
Day 1	She is transferred to her local cardiothoracic center. She remains haemodynamically stable but has further chest discomfort. She is discussed in the MDT and a decision is made for an attempt at percutaneous removal with surgical standby.
Day 2	The patient underwent successful percutaneous removal of the fragmented guidewire under GA with TEE guidance.
Day 3	The patient is discharged from hospital. She proudly takes the fragmented guidewire home with her.
Follow-up	She is reviewed in clinic where she has remained well with no further symptoms.

## Funding Support and Author Disclosures

Dr Samuel McGrath is undergoing a Clinical Research Training Fellowship (CRTF) funded by the 10.13039/501100000274British Heart Foundation (FS/CRTF/22/24187). Prof Hildick-Smith is a Proctor/Advisory to Abbott, Boston, Medtronic, and Edwards.

## Appendix

Video 1 Course of the Wire Three-dimensional computed tomography reconstruction of fragmented wire using 3mensio software.

Video 2 Short-Axis Transesophageal Echocardiography View This short-axis transesophageal echocardiography view demonstrates the wire passing through the patent foramen ovale.

Video 3 Agilis Catheter Manipulation The Agilis catheter is used to hook behind the fragmented wire.

Video 4 Snare Mechanism The snare is tightened and withdrawn into the Agilis catheter, and the catheter is moved as a whole unit.

Video 5 Degree of Tension The snare has been withdrawn into the Agilis catheter, and traction is applied to remove the fragmented wire.
